# Supplementary material for: The interactome of intact mitochondria by cross-linking mass spectrometry provides evidence for coexisting respiratory supercomplexes
Source: Mol Cell Proteomics. 2017 Dec 8;17(2):216–32. doi: 10.1074/mcp.RA117.000470 (PMC5795388; doi:10.1074/mcp.RA117.000470)
Supplement: Supplemental Data [file supp_RA117.000470_133922_0_supp_23978_fzffwf.zip › spectra_annotation/mito_DR_spectra_annotation/191-1-2-1-9-1.pdf]

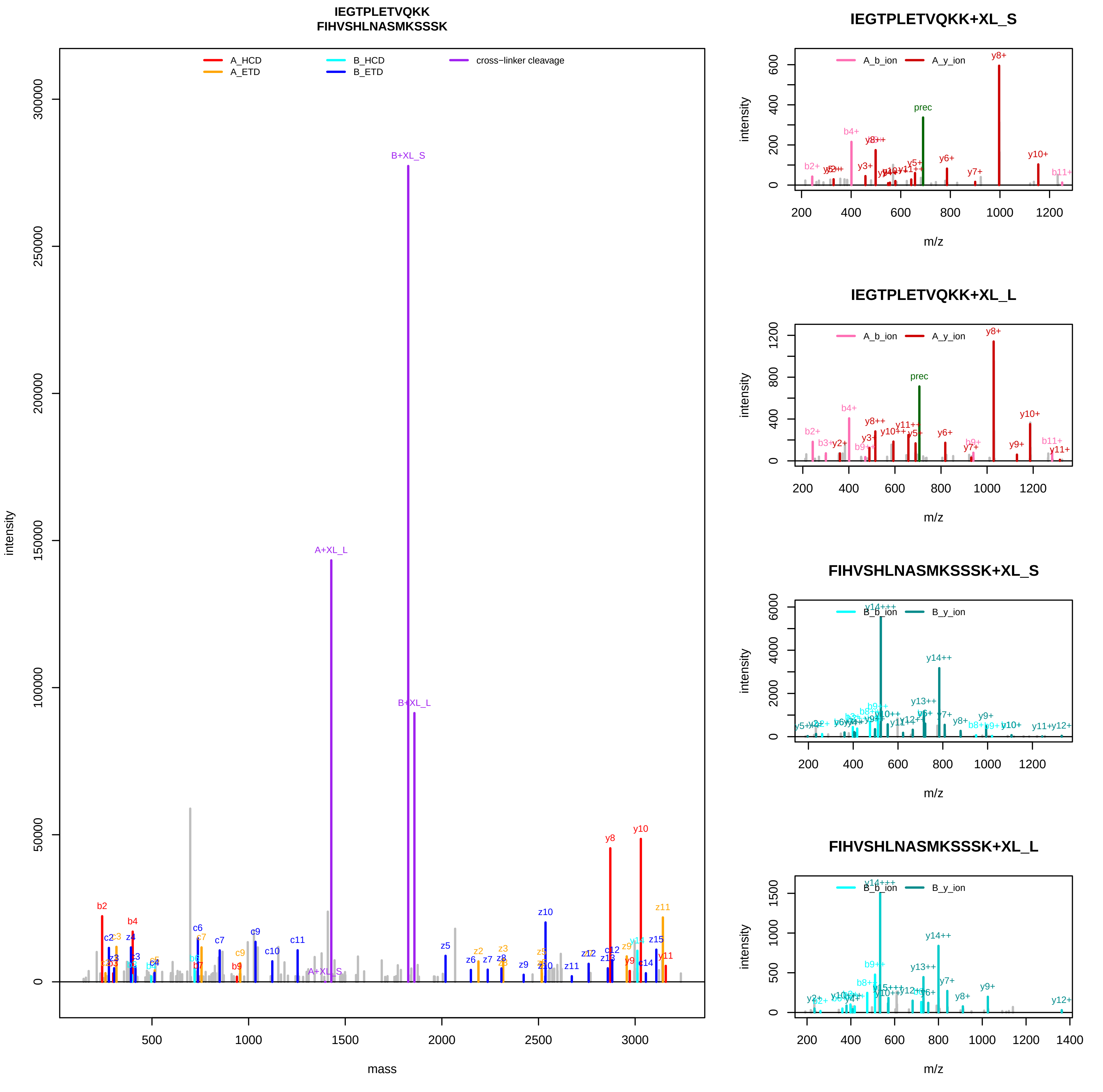

IEGTPLETVQKK
FIHVSHLNASMKSSSK
A_HCD
A_ETD
B_HCD
B_ETD
cross−linker cleavage
B+XL_S
A+XL_L
B+XL_L
A+XL_S
y8
y10
z11
z10
intensity
mass
0
50000
100000
150000
200000
250000
300000
500
1000
1500
2000
2500
3000
IEGTPLETVQKK+XL_S
A_b_ion
A_y_ion
y8+
prec
b4+
y10+
y6+
y7+
m/z
IEGTPLETVQKK+XL_L
FIHVSHLNASMKSSSK+XL_S
B_b_ion
B_y_ion
y14+++
y14++
y13++
FIHVSHLNASMKSSSK+XL_L
b9++
y7+
y9+
y12+
